# Quantifying the risk of error when interpreting funnel plots

**DOI:** 10.1186/s13643-015-0004-8

**Published:** 2015-03-11

**Authors:** Mark Simmonds

**Affiliations:** Centre for Reviews and Dissemination, University of York, Heslington, York, YO10 5DD UK

**Keywords:** Publication bias, Funnel plots, Meta-analysis, Egger’s test

## Abstract

**Background:**

Funnel plots are widely used to investigate possible publication bias in meta-analyses. There has, however, been little formal assessment of whether a visual inspection of a funnel plot is sufficient to identify publication bias.

**Methods:**

Visual assessment of bias in a funnel plot is quantified using two new statistics: the Imbalance and the Asymmetry Distance, both intended to replicate how a funnel plot is typically assessed. A simulation study was performed to assess the performance of these two statistics for identifying publication bias.

**Results:**

The two statistics both have high type I error and low statistical power, unless the number of studies in the meta-analysis is very large. These results suggest that visual inspection of a funnel plot is unlikely to lead to a valid assessment of publication bias.

**Conclusions:**

In most systematic reviews, visual inspection of a funnel plot may give a misleading impression of the presence or absence of publication bias. Formal statistical tests for bias should generally be preferred.

**Electronic supplementary material:**

The online version of this article (doi:10.1186/s13643-015-0004-8) contains supplementary material, which is available to authorized users.

## Background

Publication bias arises where studies with results that go against the prior opinion of the authors (such as when a new drug is less effective than placebo) or are not statistically significant are not published, and so are not included in a systematic review and meta-analysis, leading to biassed conclusions [1]. It is therefore important to assess whether publication bias might affect a meta-analysis. A commonly used method to assess whether this is the case is to examine a funnel plot. This is a plot of the estimate of effect size in each study against an estimate of its precision (typically its standard error) [2]. We would expect the effect estimate in large studies with high precision to be close to the true effect, while studies with lower precision will have effect estimates evenly distributed on either side of the true effect, creating a funnel-shaped plot. If there is publication bias, then studies with low precision that have negative or non-significant results will be missing from the plot because they were not published, producing a funnel plot that is asymmetric.

Identifying funnel plot asymmetry may therefore suggest the possibility of publication bias. It should be remembered, however, that funnel plot asymmetry may have causes other than publication bias: selective reporting bias, where studies are published but outcomes with results that were not statistically significant are not presented, can also result in an asymmetric funnel [3], as can cases where there are genuine differences in effect between smaller and larger studies. Of course, if there are differences in effect across studies, then a naïve meta-analysis may be inappropriate and further analyses, such as a meta-regression, may be needed.

**Figure 1:**
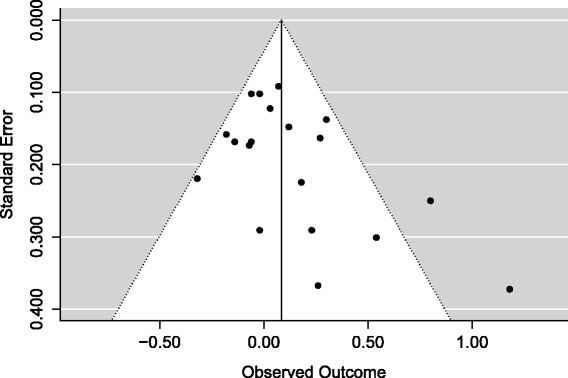
**A funnel plot for a meta-analysis of studies of teacher expectancy on the IQ of pupils.**

Figure 1 gives an example of an apparently asymmetric funnel plot from a meta-analysis of the effect of teacher expectancy on the IQ of pupils [4]. Note that there are no studies in the lower left part of the funnel. Despite this apparent asymmetry, Egger’s test for publication bias is not quite statistically significant (*P* = 0.061).

A problem with using a funnel plot to look for evidence of asymmetry and hence of publication bias is that it is purely visual. It is subjective and not a formal statistical test. This may lead to misinterpretation, particularly when there are few studies [5]. Readers may find it difficult to interpret funnel plots [6] and may be misled by the choice of axes or outcome measure [7]. Using funnel plot asymmetry to assess publication bias poses several problems including how we determine whether a plot is asymmetric, whether apparently asymmetric plots can occur by chance, how much asymmetry is required to have good evidence of bias, and how likely a plot is to be asymmetric when there is publication bias.

In this paper, we consider these issues by examining two new statistics for assessing funnel plot asymmetry: Imbalance and Asymmetry Distance. Meta-analyses with and without publication bias are simulated and the resulting funnel plots assessed for asymmetry using these new statistics. We show that apparently asymmetric funnel plots can frequently arise by chance and that even when there is considerable publication bias, funnel plots are rarely asymmetric.

## Methods

### Visual assessment of funnel plots

When examining a funnel plot for asymmetry, we might typically identify asymmetry by noticing an absence of studies on the lower corner of one side of the funnel plot and a corresponding greater number of studies in the opposite corner. This might suggest that smaller studies with unfavourable results were not published. For example, in Figure 1, there are fewer studies in the lower left corner of the funnel than the lower right corner. Hence, a simple way to assess asymmetry is to count the number of studies in each corner of the funnel plot and compare them. In Figure 1, when considering the bottom half of the funnel (where standard error is greater than 0.2), there are six studies on the lower right of the funnel but only two on the lower left side, a difference of four. For the purposes of this paper, we will call this difference in the number of studies on either side of the lower part of the funnel the ‘Imbalance’ in the funnel plot. How important any identified imbalance is must be interpreted relative to the total number of studies in the analysis.

A problem with simply counting numbers of studies on either side of the funnel is that it takes no account of how far studies are from the middle of the funnel. In Figure 1, some of the studies on the right-hand side of the funnel are considerably further from the centre line than studies on the left-hand side. To take account of this, we can consider the distance of the effect estimates from the centre line of the funnel, by comparing the difference in the sum of distances on the right side of the funnel to the sum of distances on the left side. If *θ*_i_ is the effect estimate in each study and *C* is the centre line of the funnel (typically, the summary effect estimate from a fixed-effect meta-analysis), we can thus define the ‘Asymmetry Distance’ (AD):

$$ \mathrm{AD} = \left|\frac{{\displaystyle {\sum}_{\theta_{\mathrm{i}}>C}}\left({\theta}_{\mathrm{i}}-C\right)-{\displaystyle {\sum}_{\theta_{\mathrm{i}}<C}}\left(C-{\theta}_{\mathrm{i}}\right)}{{\displaystyle {\sum}_{\mathrm{i}}}\left|{\theta}_{\mathrm{i}}-C\right|}\right|=\left|\frac{{\displaystyle {\sum}_{\mathrm{i}}}\left({\theta}_{\mathrm{i}}-C\right)}{{\displaystyle {\sum}_{\mathrm{i}}}\left|{\theta}_{\mathrm{i}}-C\right|}\right| $$

That is, the difference in distances on the two sides of the funnel dived by the total distance. The Asymmetry Distance ranges from zero (perfect symmetry) to one (maximum asymmetry, all studies on same side of the funnel). This Asymmetry Distance is harder to judge by eye than the Imbalance, but it is easily calculated using the available effect estimates, or directly from a funnel plot. In Figure 1, the Asymmetry Distance among studies with a standard error greater than 0.2 is 0.71. We will consider how to interpret this result later in this paper.

While the Imbalance and Asymmetry Distance can represent how we might visually assess a funnel plot, and provide simple means to assess funnel plot asymmetry, it is not clear how large either of these should be in order to conclude that a funnel plot is asymmetric. We investigate this in a simulation study.

### A simulation study

**Figure 2:**
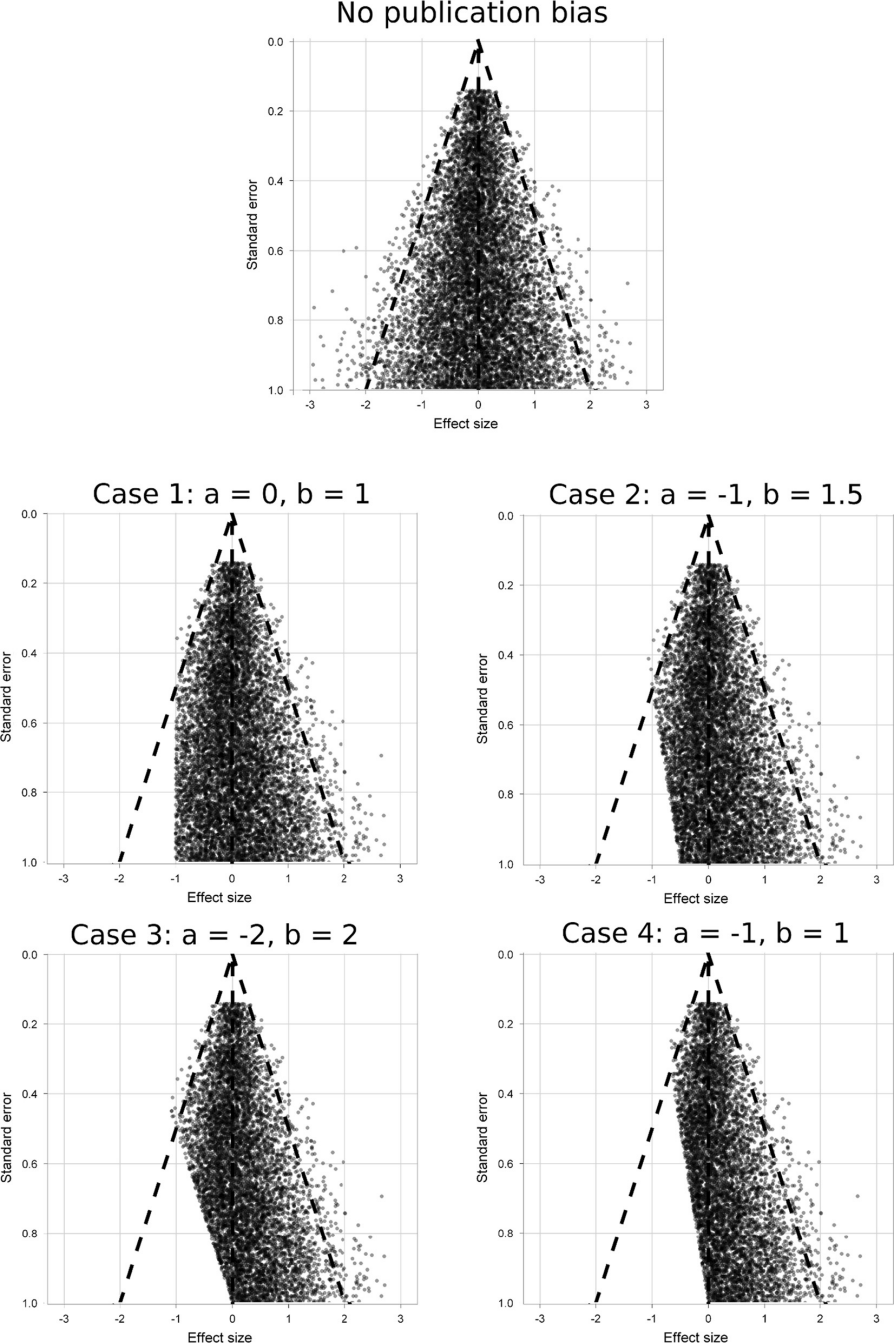
**Funnel plots of trials in the simulation study: including the case without asymmetry and four cases with asymmetry due to publication bias.**

To examine the relationship between funnel plot asymmetry and publication bias, we performed a simulation study. A total of 10,000 studies were simulated, with varying effect size *θ*_i_ and standard error *σ*_i_, by making random draws from the distributions: ${\theta}_{\mathrm{i}}\sim N\left(0,{\sigma}_{\mathrm{i}}^2\right)$ and ${\sigma}_{\mathrm{i}}^2\sim U\left[0.02,1\right]$. This choice gives a symmetric funnel plot with studies evenly distributed throughout the funnel. There is no heterogeneity in this simulated sample. The funnel plot for these 10,000 studies is shown at the top of Figure 2.

To simulate a meta-analysis, a set of *S* studies was sampled without replacement from the full set of 10,000 studies. The Imbalance and Asymmetry Distance, as defined above, were calculated for this meta-analysis of *S* studies considering only studies with *σ*_i_ ≥ 0.7 (the median standard error in the simulation), to represent assessing asymmetry based on studies at the bottom of the funnel plot. As Egger’s test for funnel plot asymmetry [8] is widely used to test for publication bias, Egger’s test was also performed to compare the visual assessments of asymmetry to a formal test. In this simulation study, meta-analyses with *S* = 5, 10, 15, 20 … up to 100 studies were considered. For each value of *S* a total of 10,000 simulated meta-analyses were tested. This simulation assessed the performance of the Imbalance and Asymmetry Distance in the absence of publication bias.

To simulate publication bias, the model of Copas and Shi was used [9]. This model has three parameters *a*, *b* and a correlation *ρ*. For each study in a simulated meta-analysis, a random variable *δ*_i_ is drawn from a standard normal distribution with the condition that $\mathrm{corr}\left(\frac{\theta_{\mathrm{i}}}{\sigma_{\mathrm{i}}},{\delta}_{\mathrm{i}}\right)=\rho$ and the study is published only if $a+\frac{b}{\sigma_{\mathrm{i}}}+{\delta}_{\mathrm{i}}>0$. The choice of *a*, *b* and *ρ* determines the degree of publication bias. Four different cases of the model with varying values of *a* and *b* were considered, as shown in Figure 2, each with *ρ* = 1, to represent differing degrees of publication bias. The parameter *ρ* can be interpreted as the probability that a study in the lower corner of the funnel is not published. Setting *ρ* = 1 therefore ensures that there are regions of the funnel with no published studies at all, so this represents fairly extreme publication bias.

The simulation process described above was repeated, but this time sampling the *S* studies for the meta-analysis from the sets with publication bias shown in Figure 2, so the simulated meta-analyses were subject to publication bias. As before, 10,000 simulated meta-analyses were examined for each *S* in 5, 10, 15… 100. The simulation process was repeated for each of the four sets of studies in Figure 2, representing four differing degrees of publication bias.

## Results

### Results without publication bias

**Figure 3:**
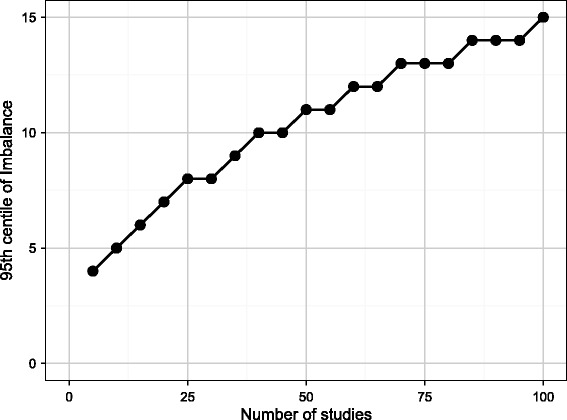
**The 95th centile of Imbalance in the absence of publication bias according to number of studies in the meta-analysis.**

We consider first the simulation study based on the symmetric funnel, without publication bias. Figure 3 shows the 95th centile of Imbalance in the simulations according to the total number of studies in the meta-analysis, where Imbalance was calculated using only studies with *σ*_i_ ≥ 0.7. These values can therefore be taken to represent the level of Imbalance needed to reject the null hypothesis of no funnel plot asymmetry. This shows that considerable funnel plot asymmetry can emerge by chance. When there are ten studies in a meta-analysis, an Imbalance of 5 is needed to conclude that there is publication bias, so all the five smallest trials must lie on the same side of the funnel. Even with 50 studies in a meta-analysis, an Imbalance of 11 is needed to reject the null hypothesis of no publication bias, so among the 25 smallest trials, the split must be at least as extreme as 18 studies on one side of the funnel to 7 on the other.

**Figure 4:**
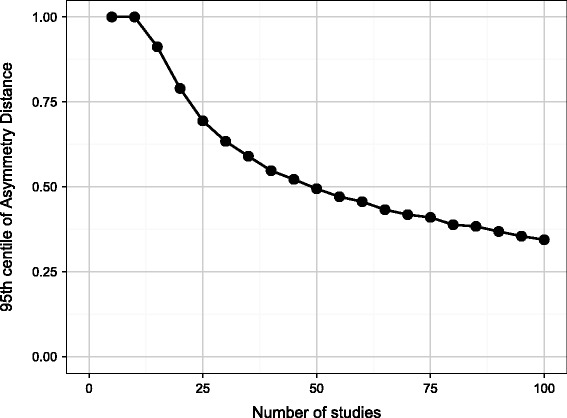
**The 95th centile of the Asymmetry Distance in the absence of publication bias according to number of studies in the meta-analysis.**

Figure 4 shows, in a similar fashion, the 95th centile for the Asymmetry Distance in the absence of funnel plot asymmetry. When there are ten or fewer studies, an Asymmetry Distance of one (the most extreme value) occurs more than 5% of the time, so no value of the Asymmetry Distance is sufficient to reject the assumption of no funnel plot asymmetry. As the number of studies increases, large distances may still occur by chance: when there are 20 studies in the meta-analysis, more than 5% of symmetric funnel plots will have an Asymmetry Distance of 0.75 or more.

These results show that the Imbalance of 4 and the Asymmetry Distance of 0.71 for Figure 1 which has 19 studies are not sufficient to conclude that there is funnel plot asymmetry as this degree of asymmetry could have arisen by chance in the absence of publication bias.

### Results with publication bias

**Figure 5:**
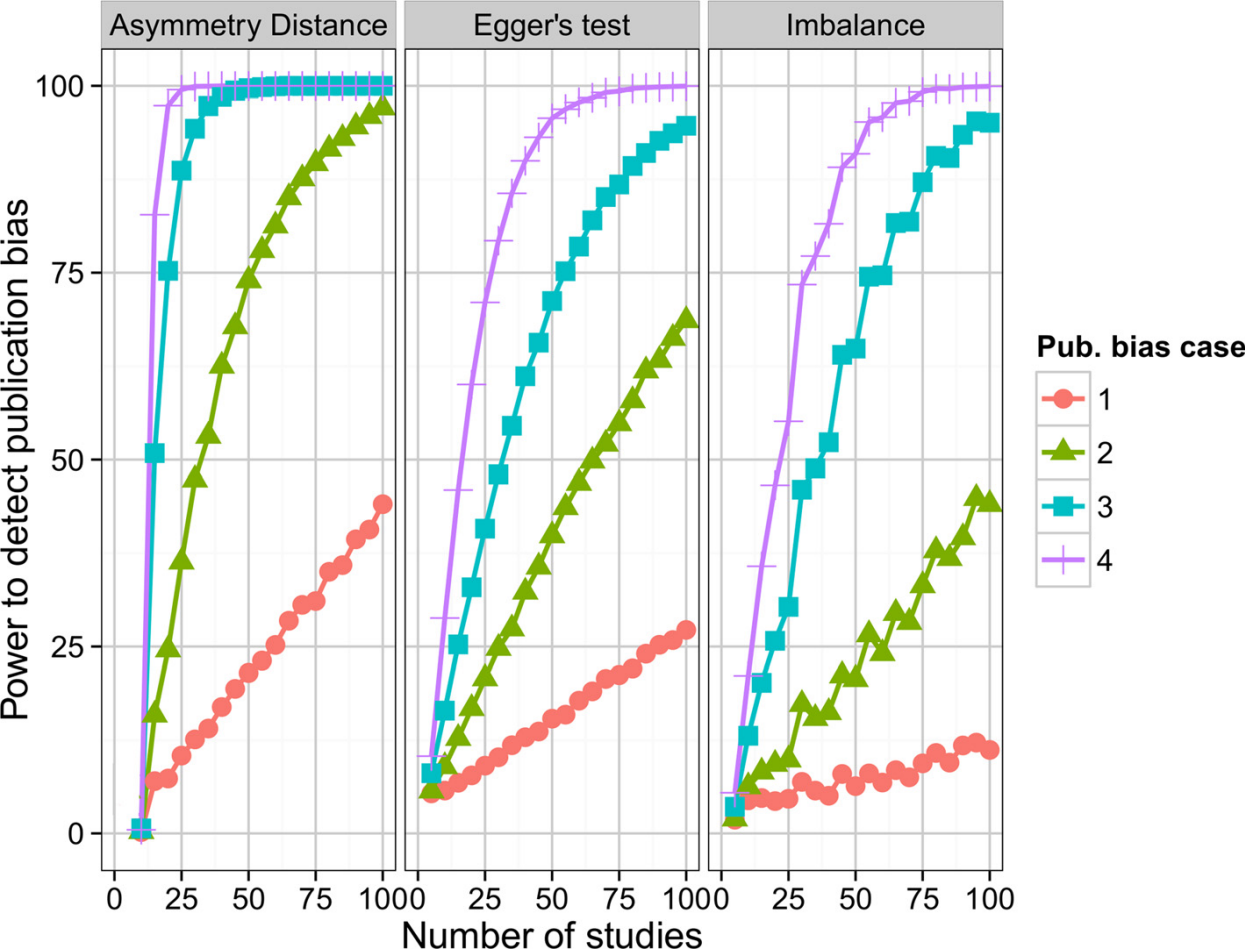
**Power to detect publication bias for Imbalance, Asymmetry Distance and Egger’s test according to number of studies and degree of publication bias.**

We next consider the simulations with publication bias. Figure 5 shows the statistical power of the Imbalance, Asymmetry Distance and Egger’s test to detect publication bias, with a 5% type I error, based on the threshold values found in Figures 3 and 4. Results are given for all four cases of publication bias shown in Figure 2 from modest bias (case 1) to extreme bias (case 4). When there are ten trials in the meta-analysis, power is low for all methods, and is zero for the Asymmetry Distance. The Imbalance has low power even with large numbers of studies and does not achieve even 50% power for the more moderate cases of publication bias. By contrast, the power using the Asymmetry Distance rises rapidly as the number of studies increases and this method is likely to detect publication bias correctly in the more extreme cases of bias if there are 20 or more studies in the meta-analysis.

This simulation confirms the low power of Egger’s test: achieving even 50% power requires either extreme publication bias or a large number of studies (for example, over 50).

The examples so far all represent fairly extreme cases of publication bias, where there were regions of the funnel plot with no publishes studies at all (Figure 2). In additional simulations studies, we found that more realistic instances of publication bias, where some studies in those regions were published (for example, by setting *ρ* = 0.8 in the Copas and Shi model, results are presented in Additional file 1), further reduce the power of both visual assessment of the funnel plot and Egger’s test to detect asymmetry.

This analysis also assumed no heterogeneity in the effect estimates across trials. We performed an additional simulation study by including a heterogeneity *τ*^2^ of 0.25 (moderate heterogeneity with *I*^2^ = 33%) when simulating study effect sizes. Including heterogeneity across the simulated studies does not affect the 95% centiles when there is no funnel plot asymmetry but does reduce the power of the funnel plot to detect asymmetry (results are presented in Additional file 2). We note that the power of Egger’s test when there is heterogeneity is lowered considerably more than the power for the Asymmetry Distance. This suggests that an inspection of the funnel plot may be preferable to Egger’s test when there is substantial heterogeneity.

## Discussion

This study has shown that a visual assessment of a funnel plot in a meta-analysis is generally a poor method of assessing whether funnel plot asymmetry or publication bias is present. Unless the number of studies in the meta-analysis is very large, apparently asymmetric funnel plots frequently occur by chance when there is no underlying asymmetry. Even when there is considerable publication bias, funnel plots lack power to detect it because most funnel plots will not appear to be particularly asymmetric.

Both the Imbalance and the Asymmetry Distance are approximations designed to give numerical values that represent how one might view a funnel plot. More sophisticated consideration of a funnel plot might improve the ability to detect funnel plot asymmetry. The problem with visually assessing a funnel plot, however, is precisely that it is not a formal statistical test. As such, there is no clear way of determining how much asymmetry is required to reject the null hypothesis of a symmetric funnel plot.

The simulation has shown that high levels of Imbalance and Asymmetry Distance are required to reject the null hypothesis of no symmetry, and when there are ten or fewer studies in the meta-analysis (very common in medical contexts), even an Asymmetry Distance of 1 (the maximum possible) is insufficient to reject the null hypothesis. It is reasonable to conclude that any funnel plot, no matter how assessed, would have to appear very asymmetric before one could be confident that the asymmetry had not arisen by chance. So there is a substantial chance of type I error when visually assessing a funnel plot. Even the funnel plot illustrated in Figure 1, which has been used elsewhere as an example of funnel plot asymmetry [4], does not in fact have sufficient asymmetry, in terms of the Imbalance or Asymmetry Distance, to reject the possibility that the asymmetry arose by chance alone, despite being on the borderline of statistical significance using Egger’s test (*P* = 0.061).

Funnel plots have many problems in addition to those identified. How the funnel plot is presented, such as which metric is used for the vertical axis, can substantially change the appearance and hence the interpretation of the plot. Other research has shown that funnel plots are often interpreted inconsistently and that researchers are generally poor at interpreting funnel plots [6]. Funnel plot asymmetry, even if it is identified, is not necessarily evidence that there is publication bias. Heterogeneity in the effect estimates can lead to asymmetry, for example, in meta-analyses of clinical trials where small trials may be targeted to individuals more likely to benefit from treatment, whereas larger trials recruit patients from a more general population. In such cases, a naïve meta-analysis may be inappropriate and further analyses, such as a meta-regression, may be needed.

## Conclusions

Presenting a funnel plot in a meta-analysis may be highly misleading, particularly when then are ten or fewer studies in the analysis, as is common in meta-analyses of clinical trials. While this paper has presented two new statistics for assessing funnel plot asymmetry, it is not proposed that they be used because of this potential for misleading interpretation. Formal statistical test of asymmetry, such as Egger’s test, should generally be preferred to funnel plots because, although such tests lack statistical power, they have appropriate type I error. Funnel plots, if they are to be presented at all, may be best used as illustrations of asymmetry when there is good evidence from a formal test that funnel plot asymmetry is present.

## Additional files

Additional file 1: Figure S1. Power to detect publication bias for Imbalance, Asymmetry Distance and Egger’s test with reduced publication bias (*ρ* = 0.8 in the Copas and Shi model). Additional file 2: Figure S2. Power to detect publication bias for Imbalance, Asymmetry Distance and Egger’s test with heterogeneity (*τ* ^2^ = 0.25).

## Abbreviations

AD: Asymmetry Distance

## References

[1] Dickersin K (1990). The existence of publication bias and risk factors for its occurence. JAMA.

[2] Light RJ, Pillemer DB (1984). Summing up: the science of reviewing research.

[3] Kirkham JJ, Dwan KM, Altman DG, Gamble C, Dodd S, Smyth R (2010). The impact of outcome reporting bias in randomised controlled trials on a cohort of systematic reviews. BMJ.

[4] Raudenbush SW (1984). Magnitude of teacher expectancy effects on pupil IQ as a function of the credibility of expectancy induction: a synthesis of findings from 18 experiments. J Educ Psychol.

[5] Lau J, Ioannidis JPA, Terrin N, Schmid CH, Olkin I (2006). The case of the misleading funnel plot. BMJ.

[6] Terrin N, Schmid CH, Lau J (2005). In an empirical evaluation of the funnel plot, researchers could not visually identify publication bias. J Clin Epidemiol.

[7] Tang J-L, Liu JLY (2000). Misleading funnel plot for detection of bias in meta-analysis. J Clin Epidemiol.

[8] Egger M, Smith GD, Schneider M, Minder C (1997). Bias in meta-analysis detected by a simple, graphical test. BMJ.

[9] Copas J, Shi JQ (2000). Meta-analysis, funnel plots and sensitivity analysis. Biostatistics.

