# Supplementary figures and images for: Quantifying the risk of error when interpreting funnel plots
Source: Syst Rev. 2015 Mar 11;4:24. doi: 10.1186/s13643-015-0004-8 (PMC4460648; doi:10.1186/s13643-015-0004-8)

Power to detect publication bias

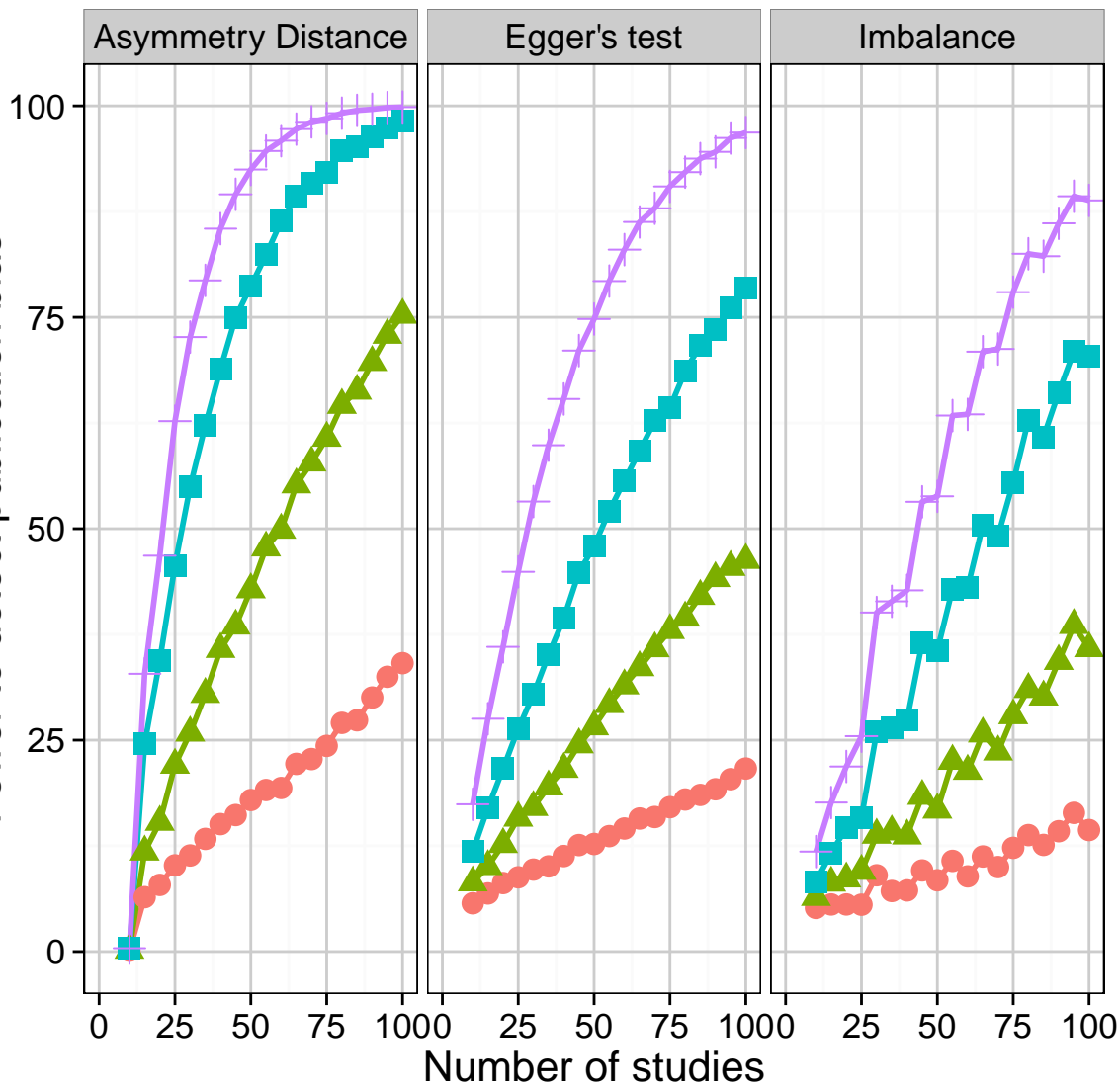

Supplement: Additional file 1: Figure S1. — Power to detect publication bias for Imbalance, Asymmetry Distance and Egger’s test with reduced publication bias (ρ = 0.8 in the Copas and Shi model). [file 13643_2015_4_MOESM1_ESM.pdf]

Power to detect publication bias

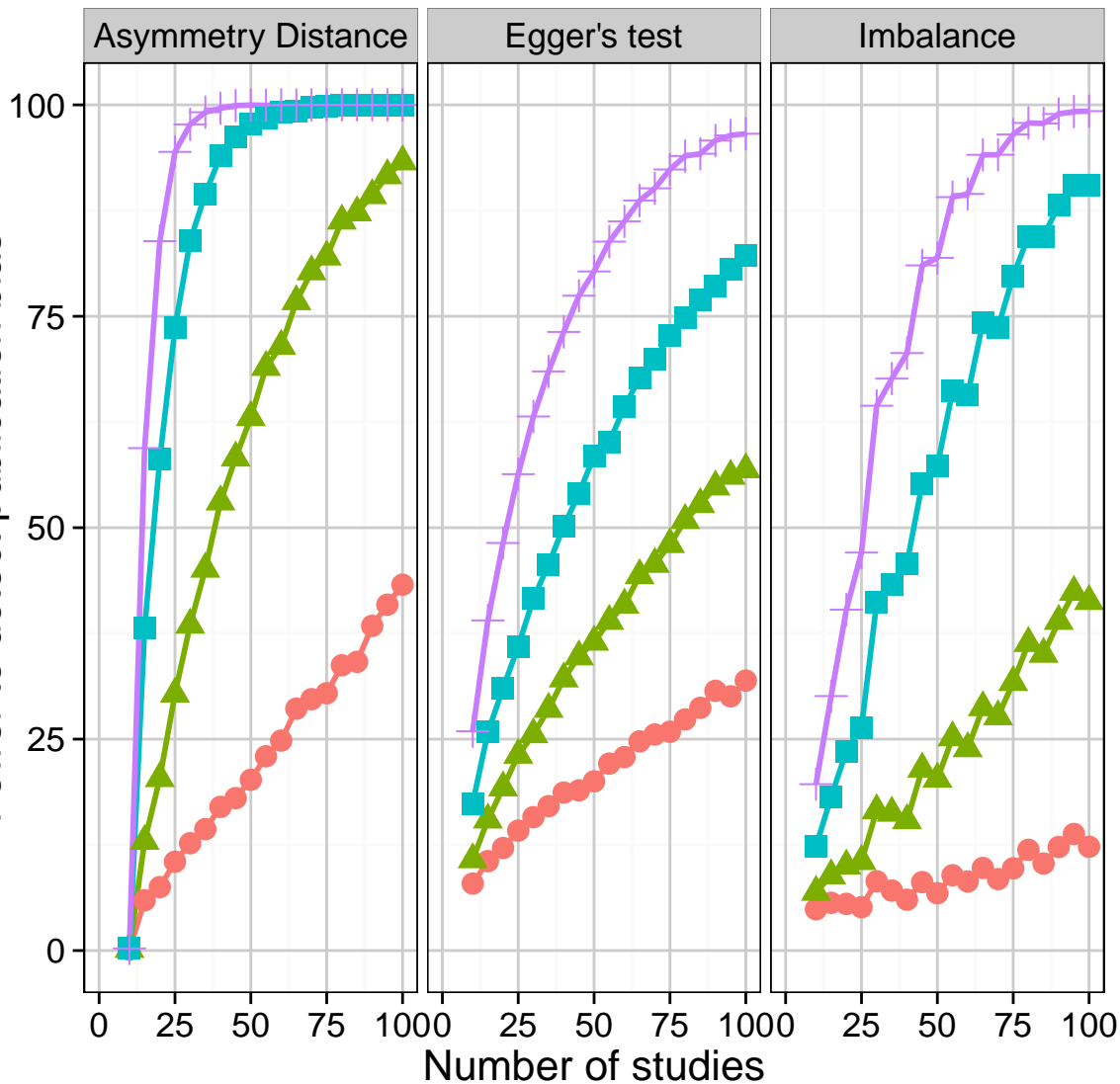

Supplement: Additional file 2: Figure S2. — Power to detect publication bias for Imbalance, Asymmetry Distance and Egger’s test with heterogeneity (τ 2 = 0.25). [file 13643_2015_4_MOESM2_ESM.pdf]
